# Integrating Gut Microbiome and Metabolome to Gain Insight into Metabolic Dysfunction-Associated Steatotic Liver Disease

**DOI:** 10.1016/j.gastha.2025.100783

**Published:** 2025-08-29

**Authors:** Xiaofang Zhang, Jinluan Chen, Laurens A. van Kleef, Carolina Medina-Gomez, Joyce B.J. van Meurs, M. Arfan Ikram, Robert Kraaij, Willem Pieter Brouwer, Robert J. de Knegt, Shahzad Ahmad, Mohsen Ghanbari

**Affiliations:** 1 Department of Epidemiology, Erasmus MC University Medical Center, Rotterdam, The Netherlands; 2 Department of Internal Medicine, Erasmus MC University Medical Center, Rotterdam, The Netherlands; 3 Department of Gastroenterology and Hepatology, Erasmus MC University Medical Center, Rotterdam, The Netherlands

**Keywords:** Hepatic steatosis, MASLD, Gut microbiota, Plasma metabolome, Metabolomics

## Abstract

**Background and Aims:**

Recent evidence has implicated the gut microbiome in the regulation of host metabolites and the pathogenesis of metabolic dysfunction–associated steatotic liver disease (MASLD). However, the role of gut microbiome and its associated metabolites as regulators and/or modulators in MASLD remains understudied. We aimed to investigate the gut microbiome diversity and composition, its interaction with plasma metabolites, and MASLD risk using population-level data.

**Methods:**

We combined 16S rRNA sequencing of stool samples with a wide range of plasma metabolites measured by the Metabolon HD4 platform, and MASLD diagnosed by abdominal ultrasound in the Rotterdam Study cohort at baseline, between 2012 and 2014. We assessed the relationship of gut microbiome and metabolites with MASLD separately. Then, the metabolic profile as exposure was obtained through multivariate linear or logistic regression models for MASLD, liver fibrosis, alpha diversity, and MASLD-associated genera.

**Results:**

Our gut microbiome analysis showed that alpha diversity was lower in MASLD individuals (Shannon: *P* value = 2.06 × 10^−6^), beta diversity differed across MASLD strata (R^2^ = 0.00158, *P* value <.001), and *Lachnospiraceae UCG-010* (odds ratio = 1.14 (1.06–1.23), area under the curve = 0.7807)) was significantly associated with MASLD, after multiple testing correction (q value<0.05). The metabolomics analysis showed 160 out of the 991 studied metabolites to be significantly associated with MASLD (q value<0.05). Moreover, 57 of the identified metabolites were significantly correlated with gut microbiome at the genus level in individuals with MASLD.

**Conclusion:**

This study indicates alterations in several plasma metabolites to be linked to gut microbiome in MASLD. These results lay the ground for future studies to better understand the host-gut microbiota metabolic interactions in the development of MASLD.

## Graphical abstract

**Figure:**
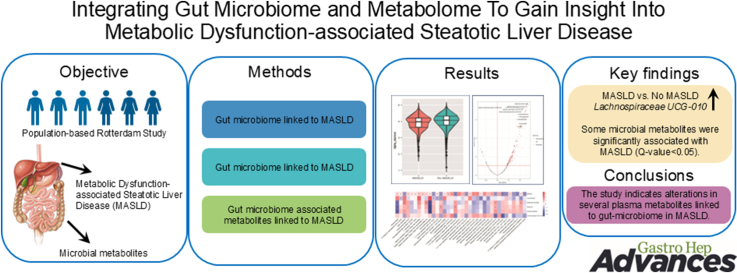

## Introduction

Metabolic dysfunction–associated steatotic liver disease (MASLD), has become a prominent global health issue, affecting over 30% of the world’s adult population.^1^^,^^2^ MASLD is characterized by excess accumulation of triglyceride in the liver. It ranges from simple fat buildup (steatosis) to the more severe form, metabolic dysfunction–associated steatohepatitis, which can lead to fibrosis, cirrhosis and liver cancer.^3^ The severity of MASLD is mediated by factors that include the gut microbiome and gut-derived metabolites.^4^^,^^5^

There is growing evidence of the role of the gut microbiome in the regulation of host metabolites that can enter into systemic circulation and influence the risk and pathogenesis of MASLD.^4^ One such instance, embedded in the Rotterdam Study cohort using 16S rRNA gene sequencing gut microbiome and the high-throughput proton nuclear magnetic resonance metabolomics data, have shown branched-chain amino acids (BCAAs) and glycoprotein acetyls of metabolic profiles and alpha diversity indices show opposite directions associated with steatotic liver disease.^5^ Additionally, recent evidence propose that trimethylamine-N-oxide (TMAO), a metabolite produced by bacteria cometabolism of choline and L-carnitine from the diet, can aggravate liver steatosis through bile acid metabolism in MASLD. Furthermore, studies have reported associations between specific bacteria, e.g. *Ruminococcus torques* (*R. torques) group*,^6^ *Veillonellaceae*^7^ and specific metabolites, eg leucine,^8^ glycoprotein acetyls,^5^ and MASLD in humans, although results have varied due to factors such as small sample sizes, diverse assessment methods for the gut microbiome and metabolites, and control for confounding variables.

Given the close association of gut microbiome and microbiome-derived metabolites on MASLD, we systematically analyzed the gut microbiome along with a wide range of plasma metabolites in the prospective population-based Rotterdam Study cohort. Our objectives were to characterize the gut microbiome composition in individuals in MASLD, explore associations between plasma metabolites and MASLD, and identify gut bacteria-associated metabolites linked to the presence of MASLD and liver fibrosis in this population-based cohort study.

## Patients and Methods

### Study Population

**Figure 1:**
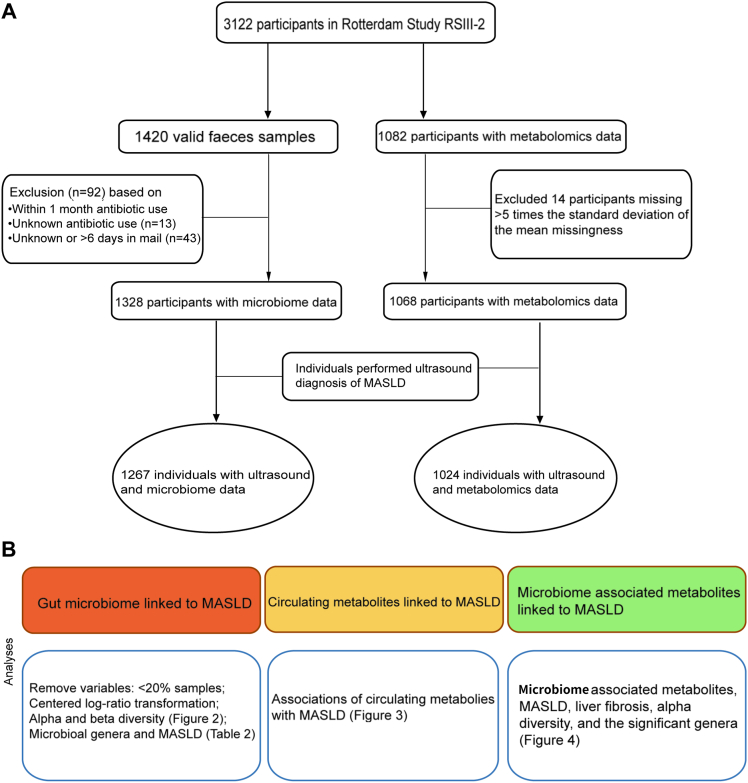
Overview of the study population and conducted analyses. (A) The flowchart of selecting the study population. (B) Overview of the analyses performed.

The Rotterdam Study is a large, prospective, population-based cohort study of individuals aged ≥40 years living in the Ommoord region of Rotterdam, the Netherlands. The objectives, study design, and recent findings of the Rotterdam Study have been described in detail elsewhere.^9^ This cross-sectional study was performed in the second visit of the third cohort (RS-III-2), in which participants were asked to collect fecal and blood samples for microbiome and metabolomics analyses, and were invited to perform an abdominal ultrasound for the diagnosis of MASLD. Inclusion criteria for participation in the current study were availability of metabolomics, gut microbiome, and ultrasound data. A total of 1420 individuals had gut microbiome data. We excluded 92 participants who had used antibiotics within 1 month before stool sample collection or for whom antibiotic use information was not available, as well as those whose stool sample was in mails more than 6 days or unknown, leaving 1328 individuals. Then, we excluded 61 participants who did not have ultrasound data. This left 1267 participants for gut microbiome and MASLD analysis. From the same visit, a total of 1082 participants had metabolomics data. We excluded 14 participants with missing metabolomics data on >5 times the standard deviation of the mean missingness of the total participants, and 44 individuals lacking ultrasound data. This left 1024 individuals for our analysis to investigate the associations between metabolites and MASLD (Figure 1A).

### Fecal Samples and 16S Ribosomal RNA Sequencing

Participants were instructed to collect fecal samples in sterile tubes at home in a standardized fashion and sent through regular email to the Erasmus Medical Center. Upon arrival, samples were recorded and stored at −20 °C. An aliquot of approximately 300 mg was homogenized in stool stabilizing buffer according to the manufacturer’s protocol (Arrow Stool DNA; Isogen Life Science, De Meern, the Netherlands). Homogenized samples were bead in lysing Matrix B tubes containing 0.1 mm silica beads (MP Biomedicals®, LLC, Bio Connect Life Sciences BV, Huissen, the Netherlands). Samples were then centrifuged and the supernatant was subjected to automated DNA isolation according to the manufacturer’s protocol (Arrow; DiaSorin S.P.A., Saluggia, Italy). Isolated DNA was then stored at −20 °C.

The process of gut microbiome dataset generation in the Rotterdam Study has been described in detail elsewhere.^10^ Briefly, 16S rRNA gene amplification of variable regions 3 and 4 was used to sequence all samples by the Illumina MiSeq® platform v3. Phylogenetic multisample profiling was performed using a pipeline based on Divisive Amplicon Denoising Algorithm 2, an opensource R package (https://github.com/benjjneb/dada2).^11^ Taxonomy assignment using the naïve Bayes classifier. Bayesian recursive dyadic partitioning algorithm trained on the SILVA version 138.1 microbial database.^12^ Moreover, Amplicon Sequence Variant (ASV) table, taxonomy table, and metadata were then combined into a phyloseq object for analysis.^13^

### Plasma Metabolomics Profiling

We profiled blood plasma samples from 1082 participants of the RS-III-2 cohort using the untargeted Metabolon HD4 platform by Ultrahigh Performance Liquid ChromatographyTandem Mass Spectroscopy. In total, 1387 metabolites from various biochemical pathways (eg lipids, amino acids) were quantified based on their relative abundances, calculated using the area under the curve (AUC) of detected peaks. To correct for inter-day instrument variation, a block correction was applied by scaling the median value of each metabolite per run-day to 1.00, with all other values adjusted proportionally. A detailed description of the Metabolon HD4 analytical methods and data extraction procedures is available elsewhere and is briefly summarized in Ahmad S *et.al*.^14^ Before performing the imputation, we excluded 14 participants with missingness greater than 5 times the standard deviation of the mean missingness in all participants, resulting in a sample size of 1082. Metabolites with more than 70% missing values were excluded. We further excluded metabolites with a coefficient of variance greater than 30%, leaving 1111 metabolites after the quality control. For this study, we included 991 metabolites with ≤30% missingness. After log2 transformation, missing values were imputed using the K-nearest neighbors method, which has previously been shown to perform robustly in metabolomics data.^15^

TMAO and its precursors betaine, carnitine, deoxycarnitine, and choline were quantified in plasma samples of the same participates by using liquid chromatography tandem mass spectrometry method. Detailed description of the method can be found elsewhere.^14^

### Hepatic Steatosis and Fibrosis Assessment

Abdominal ultrasound was performed by a certified and experienced technician using the Hitachi HI VISION 900 (Tokyo, Japan). Diagnosis of hepatic steatosis was determined dichotomously as the presence of hyperechogenic liver parenchyma according to the protocol by Hamaguchi et al.^16^ MASLD was characterized by patients with hepatic steatosis and at least 1 of 5 cardiometabolic risk factors (adult can be found elsewhere).^3^

Liver stiffness measurements (LSMs) were carried out in participants using transient elastography (FibroScan®, EchoSens, Paris, France). According to the Boursier criteria,^17^ measurements were considered unreliable and were discarded in case of an interquartile range >30%, together with an LSM ≥7.1 kilopascal (kPa). Subsequently, liver fibrosis was defined as LSM ≥8.0 kPa.^18^

### Assessment of Covariates

All covariates used in this study were derived from the extensive home interview, by drawing fasting blood samples, and by automated linkage with a local pharmacy, general practitioner, and hospital. Height and weight were measured, and the body mass index (BMI) [(weight in kg)/(height in m)^2^] was calculated. Smoking status was categorized into never, current or former. Type 2 diabetes was defined according to recent World Health Organization guidelines as fasting blood glucose ≥7.0 mmol/L or nonfasting blood glucose between ≥11.1 mmol/L or the use of antidiabetic medications and was validated based on health records from general practitioners or hospitals. We used the metabolic equivalent of task to quantify activity intensity based on an adapted version of the Zutphen Physical Activity Questionnaire.^19^ Semiquantitative (389-item) food frequency questionnaires were used to extract information on alcohol consumption, energy intake and dietary quality score.^20^^,^^21^ The dietary quality score, ranging from 0 to 14, reflects adherence to the 2015 Dutch Dietary Guidelines. Alcohol consumption was assessed in grams of ethanol per day. Information on medication use including lipid-lowering drugs and antidiabetic medications was retrieved using automated linkage with the local pharmacy in which 98% of the participants were registered.

### Statistical Analyses

Descriptive statistics were used to describe study participants characteristics. Continuous data were described with normally distributed variables provided as mean (standard deviation) and non-normally distributed variables as median with 25th–75th percentile. Categorical data were presented as percentage. Missing data in the covariates (ranging from 0.24% to 24%) were handled using multiple imputation. We created 50 imputed datasets using the R-package MICE 3.13.0. Analyses were conducted on each dataset, and the results were combined using Rubin’s rules to account for the uncertainty in the imputed values. All statistical analyses were performed using R statistical language (version 4.2.3).

For gut microbiome analysis, we adjusted a full model that included the following cofactors: age, sex and technical covariates (DNA isolation batch and time in the mail), BMI, smoking status (never, current or former), daily alcohol consumption (gram/day), energy intake (kcal/day), Dutch diet quality score, physical activity (METeqh/wk), and antibiotic use (yes/no). Moreover, for metabolites analysis, potential confounders included age, sex, BMI, smoking status (never, current or former), daily alcohol consumption (gram/day), and lipid-lowering medication use (yes/no).

### Association of the gut microbiome with MASLD

The mean relative abundance of all phyla was calculated. In addition, we calculated the alpha diversity indices (Observed richness, Shannon, and Inverse Simpson indexes) and log-transformed Firmicutes to Bacteroides ratio (FBR). Difference in the above measurements by MASLD status were visualized using violin plots and evaluated using Wilcoxon rank test with continuity correction. Moreover, multivariable logistic regression models were used to assess the association of alpha diversity indices and log-transformed FBR with MASLD.

To estimate the beta diversity, a principal component analysis was performed by calculating Euclidean distances metric based on the centered log-ratio (CLR) transformed ASV count data by using the “phyloseq::ordinate” function. We used the “microbiome” package for CLR transformation, a pseudo count of 1 was then applied across the dataset to allow for log transformation. To visualize sample distances, we extracted the first 2 principal components that explained the most variation of gut microbiota composition based on CLR-transformed ASV abundances. Moreover, to evaluate the percentage of explained gut microbiome variability by MASLD, we used permutation multivariate analysis of variance (PERMANOVA, n = 999), by using the function “adonis2” from the R package “vegan”.

Furthermore, to investigate the association between the gut microbiome and MASLD, multivariable logistic regression was applied to the CLR-transformed counts of the taxonomic table at genus level. The analysis was performed for genera present in more than 20% of the samples to reduce the possibility of chance findings.^22^

### Associations of circulating metabolites with MASLD

Prior to the analysis, we performed scaling of the metabolites to mean zero and the metabolites were expressed in standard deviations. Then, multivariate logistic regression models adjusting for potential confounders were used to investigate the association between circulating metabolites and MASLD.

### Microbiota-related metabolites linked to MASLD

We first extracted plasma metabolites present in the Metabolon HD4 platform that were associated with gut microbiota at the genus level in the same population.^14^ Then, we examined the association of microbiota-related metabolites with MASLD. Additionally, we checked whether a known gut microbiota-derived metabolite TMAO and its precursors (betaine, carnitine, choline, and deoxycarnitine) were associated with MASLD. Moreover, we investigated the association of MASLD related microbial metabolites (as exposure) with liver fibrosis (as outcome) using multivariate logistic regression.

In the next step, we also assessed the associations of MASLD-related microbial metabolites (as exposure) with alpha diversity and significant MASLD-associated genera. The overall analysis scheme is depicted in Figure 1B.

## Results

### Participants Characteristics

The flowchart of participants selection and exclusion and the main analyses performed in the study are depicted in Figure 1A. The final study population consisted of 1267 participants for gut-microbiome analysis and 1024 participants for the metabolomics analysis. The details on demographics, lifestyle, cardiometabolic risk factor, medication use, microbial diversity and technical covariates of the study participants are presented in Table A1. The characteristics of 836 participants with metabolomics, gut-microbiome, and ultrasound data with information on the MASLD strata are presented in Table 1. Among them, 31.1% had MASLD, which carried a higher risk of cardiometabolic disease than those without MASLD.

**Table 1:** Characteristics of the Study Participants

Characteristic	No MASLD, n = 576 (68.9%)	MASLD, n = 260 (31.1%)	*P* value^a^
Liver fibrosis, n (%)^b^	20 (3.5)	20 (7.7)	.013
Age, y	65.6 (6.3)	62.8 (5.2)	.19
Female, n (%)	342 (59.4)	122 (46.9)	.0010
Lifestyle behaviors			
Current/former smoking, n (%)	384 (66.7)	179 (68.8)	.59
Alcohol intake, gram/day	0.64 (0.16–0.86)	6.4 (0.5–8.6)	.012
Total energy intake (kcal/d)	2286 (1921–2764)	2283 (1810–2813)	.83
Physical activity (METeqh/wk)	50.2 (22.6–84.8)	42.5 (20.4–77.1)	.093
Dutch diet quality score^c^	7.0 (6.0–8.0)	7.0 (6.0–8.0)	.06
Cardiometabolic risk factors			
BMI	26.1 (3.6)	29.9 (4.3)	2.2 × 10^−16^
Waist circumference			
Male	94.2 (87.9–101.9)	104.5 (97.6–111.6)	1.38 × 10^−15^
Female	84.6 (77.8–92.3)	96.2 (89.3–103.1)	2.20 × 10^−16^
Type 2 diabetes, n (%)	32 (5.6)	54 (20.8)	4.71 × 10^−11^
Antidiabetics therapy, n (%)	17 (3.0)	32 (12.3)	2.32 × 10^−7^
Hypertension, n (%)	321 (55.7)	186 (71.5)	2.09 × 10^−5^
Triglycerides ≥1.7 mmol/L, n (%)	110 (19.1)	114 (43.8)	2.20 × 10^−16^
HDL-cholesterol			
Male ≤1.0 mmol/L, n (%)	29 (5.0)	47 (18.1)	2.59 × 10^−10^
Female ≤1.3 mmol/L, n (%)	46 (8.0)	45 (17.3)	8.83 × 10^−16^
Lipid lowering treatment, n (%)	135 (23.4)	83 (31.9)	.012
Medication using			
Antibiotic use, n (%)			
No	477 (82.8)	219 (84.2)	.68
1–3 mo	32 (5.6)	16 (6.2)	.85
4–12 mo	67 (11.6)	25 (9.6)	.46
Microbial diversity			
Shannon index	4.1 (3.8–4.3)	4.0 (3.7–4.2)	2.79 × 10^−5^
Inverse Simpson index	33.2 (22.5–42.9)	28.6 (20.5–37.9)	9.93 × 10^−8^
Observed richness index	156.5 (117.0–198.0)	144.0 (107.8–183.5)	5.81 × 10^−4^
Technical covariates			
Time in the mail, n (%)			
0–3 d	575 (99.8)	259 (99.6)	.45
4–6 d	1 (0.2)	1 (0.4)	.32
DNA isolation batch 0/1^b^	450/126	192/68	.20

Value is presented as mean (standard deviation), sample sizes (%), or median (interquartile range).

HDL, high density lipoprotein.

a *P* value is assessed using analysis of variance, Kruskall–Wallis, or chi-squared tests.

b Liver fibrosis (LSMs ≥8.0 kPa): A group N = 1116, B group N = 893, C group N = 836 reliable measurements.

c Theoretical range 0–14, where a higher score is better adherence to the Dutch dietary guidelines.

### Reduced Microbiome Alpha Diversity in MASLD Individuals

**Figure 2:**
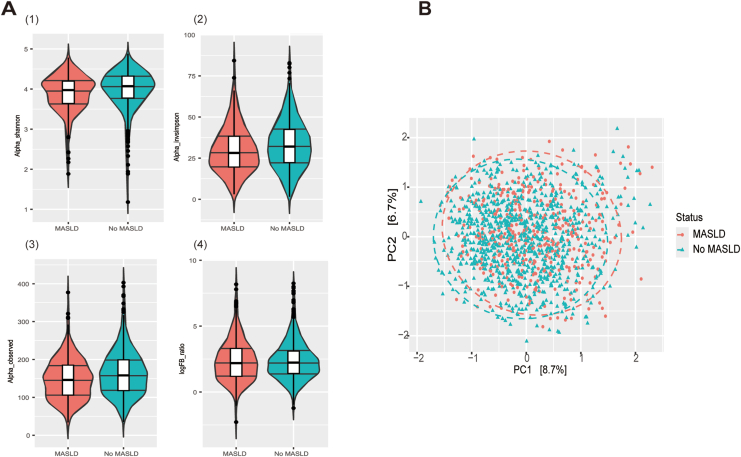
Microbiome diversity analysis. (A) Visualization of alpha diversity and FBR ratio using violin plots across MASLD strata. Violin plots of the distribution of alpha diversity, as assessed by (A) Shannon index, (B) Inverse Simpson, (C) Observed richness indexes, and (D) Log-transformed FBR. Box elements show the median, upper, and lower quartiles. ∗*P* value<.05, ∗∗*P* value<.01, ∗∗∗*P* value<.001. The difference between MASLD strata was assessed using Wilcoxon rank test with continuity correction as follows: (A) Shannon: *P* value = 2.06 × 10-6; (B) Inverse Simpson: *P* value = 2.20 × 10–16; (C) Observed richness: *P* value = 3.38 × 10-5; (D) Log-transformed FBR: *P* value = .85. (B) Principal component analysis based on ASV-level microbial composition of 1267 samples (PC1 = 8.7%, PC2 = 6.7%). PCA on CLR-transformed genus level read counts. The red dots reflect those with MASLD and the blue dots reflect those with no MASLD. Both plots show the group centroids. For the statistical analyses, we used Permutational multivariate analysis of variance (PERMANOVA) tests of associations between MASLD and microbiota composition: R^2^ = 0.00158, *P* value <.001. The model was adjusted for age, sex, time in the mail, DNA isolation Batch, BMI, smoking status, daily alcohol consumption, energy intake, physical activity, and antibiotic use. PCA, principal component analysis.

We identified 10 different phyla in our study. Figure A1 illustrates the pie chart of mean relative abundance per phylum expressed as percentages, along with the box plot comparing the relative abundance of microbial phyla in individuals with MASLD vs those without MASLD. Detailed data are presented in Table A2. The most abundant phylum was Firmicutes (79.77%), followed by Bacteroidetes (13.00%), Proteobacteria (4.13%), Actinobacteria (2.58%), Verrucomicrobiota (0.33%) and other phyla (0.19%). Alpha diversity was significantly lower in individuals with MASLD as compared to individuals without MASLD (Figure 2A Shannon: *P* value = 2.06 × 10^-6^; Inverse Simpson: *P* value = 2.20 × 10^-16^; Observed richness: *P* value = 3.38 × 10^-5^), and the difference persisted after adjusting for covariates (Table A3). There was no statistically significant difference in FBR between individuals (Figure 2A *P* value = .85).

### Microbiota Beta Dissimilarity Between Individuals with and without MASLD

Beta diversity based principal component analysis plots of human gut microbiomes of the 1267 participants included in the microbiome analyses is shown in Figure 2B. PC1 and PC2 axes explain 8.7% and 6.7% of microbiome variability, respectively. After applying the fully adjusted model, MASLD strata explained a significant amount of microbiome pattern variability (R^2^ = 0.00158, *P* value<.001).

### Associations Between Gut Microbiome and MASLD

In total, 90 genera were present in more than 20% of the included samples. We found that *Lachnospiraceae UCG-010* (odds ratio = 1.14 (1.06–1.23), AUC = 0.7807) was significantly associated with MASLD in our population, after false discovery rate (FDR) correction (q value< 0.05, Table 2). Moreover, 17 other genera were nominally associated (*P* value< .05) with MASLD and AUC were more than 0.75. Furthermore, we reviewed and summarized the functional roles and disease associations of the 17 identified genera based on previous literature, as presented in Table A4.

**Table 2:** Seventeen Gut-Microbial Genera Associated With MASLD

Genus	Median abundance of genera		Odds ratio (95% CI)	*P* value	Q value	AUC
	No MASLD, n = 877	MASLD, n = 390				
*Lachnospiraceae UCG-010*	**0 (0-18.0)**	**0 (0-26.0)**	**1.14 (1.06-1.23)**	**.0005**	**0.0410**	**0.7807**
*Bilophila*	0 (0–3.0)	0 (0–5.0)	1.18 (1.06–1.30)	.0022	0.0778	0.7785
*Adlercreutzia*	0 (0-0)	0 (0–11.0)	1.14 (1.04–1.24)	.0031	0.0778	0.7779
*Clostridium sensu stricto 1*	76.0 (0.0–323.0)	29.0 (0.0–194.0)	0.92 (0.87–0.97)	.0034	0.0778	0.7786
*Parasutterella*	0 (0–9.0)	0 (0–13.0)	1.12 (1.03–1.21)	.0058	0.1063	0.7774
*R. torques group*	282.0 (136.0–638.0)	351.5 (155.5–712.2)	1.15 (1.04–1.27)	.0085	0.1285	0.7781
*Christensenellaceae R-7 group*	292.0 (61.0–806.0)	161.5 (28.0–519.5)	0.91 (0.85–0.98)	.0148	0.1928	0.7773
*Eubacterium ruminantium group*	0 (0–84.0)	0 (0–30.75)	0.94 (0.88–0.99)	.0217	0.2402	0.7782
*CAG-352*	0 (0–55.0)	429.3 (0.0–157.2)	1.06 (1.01–1.11)	.0264	0.2402	0.7781
*Eubacterium xylanophilum group*	32.0 (0–97.0)	0 (0–72.75)	0.92 (0.86–0.99)	.0288	0.2402	0.7780
*Dorea*	394.0 (199.0–716.0)	475.0 (222.5–979.0)	1.12 (1.01–1.25)	.0306	0.2402	0.7766
*Flavonifractor*	0 (0-0)	0 (0–7.0)	1.10 (1.011.20)	.0334	0.2402	0.7769
*Fusicatenibacter*	431.0 (189.0–799.0)	401.0 (164.8–848.2)	0.92 (0.84–0.99)	.0343	0.2402	0.7781
*Agathobacter*	766 (322–1553)	1059.5 (480.5–1952.5)	1.10 (1.01–1.20)	.0381	0.2402	0.7763
*Romboutsia*	118.0 (13.0–374.0)	65.5 (0.0–269.0)	0.94 (0.89–1.00)	.0396	0.2402	0.7757
*Turicibacter*	0 (0–42.0)	0 (0–18.0)	0.93 (0.87–1.00)	.0461	0.2569	0.7760
*Lachnospiraceae AC2044 group*	0 (0–8.0)	0 (0-0)	0.91 (0.83–1.00)	.0496	0.2569	0.7764

The table shows results of the microbial genera significantly associated with MASLD., Models were adjusted for demographic, lifestyle, dietary; DNA, isolation batch, time in the mail, age, sex; BMI, smoking, alcohol intake, energy intake, Dutch diet quality score, physical activity, and antibiotic use. Abundance medians (P25-P75) of ASV, count data are given for the group with MASLD, and without MASLD., Results are given Odds Ratio, 95% confidence interval, nominal *P* value, and q value-corrected for multiple comparisons using Benjamini-Hochberg. The Benjamini-Hochberg corrected significance threshold is q value<0.05 and the significant threshold is bold.

### Association Between Plasma Metabolites and MASLD

**Figure 3:**
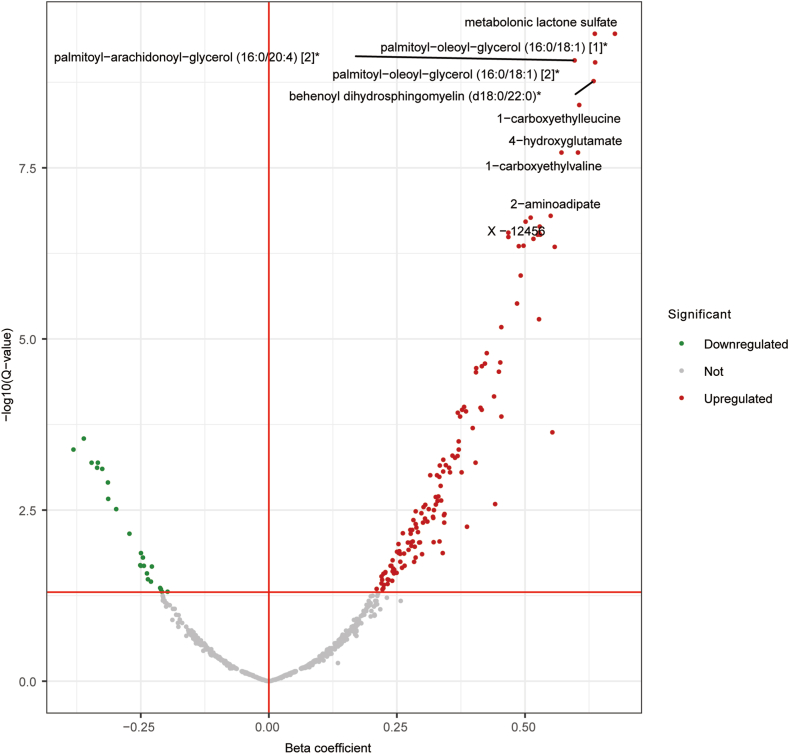
Volcano plot showing the association between circulating metabolites and MASLD. Association results after adjusted for age, sex, BMI, smoking status, alcohol consumption, and lipid-lowering medication use. The name of top 10 metabolites significantly associated with MASLD are mentioned in the figure. The red dots indicate metabolites positively significantly associated at q value<0.05; the green dots indicate metabolites negatively significantly associated, the gray dots indicate metabolites with no significant association. The red vertical lines for beta coefficient thresholds, and the red horizontal line for the q value threshold. We corrected for multiple testing using Benjamini-Hochberg significant q values, and the q value threshold is FDR corrected q value<0.05.

In the multivariate logistic regression analysis, 160 out of the 991 studied metabolites were significantly associated with MASLD (q value<0.05). A volcano plot showing the 136 metabolites positively associated with MASLD and the 24 metabolites negatively associated, is depicted in Figure 3. The details of the results are shown in Table A5.

## Gut Microbiota-Related Metabolites in Association with MASLD

We matched the MASLD-associated metabolites with the gut microbial associated metabolites using linear regression adjusting for age, sex, BMI, medication use, and technical covariates. A total of 57 metabolites out of the 160 MASLD-associated metabolites were also associated with gut microbiota at the FDR corrected level (q value< 0.05, Table A6). In addition, we found that gut microbial-related TMAO substrate metabolites, ie betaine and deoxycarnitine, were significantly associated with MASLD, at the FDR-corrected level (q value< 0.05, Table A7). Moreover, we conducted a sensitivity analysis with additional adjustment for energy intake and dietary quality scores. The associations of betaine and deoxycarnitine with MASLD remained significant after FDR correction (q value < 0.05; Table A8).

**Figure 4:**
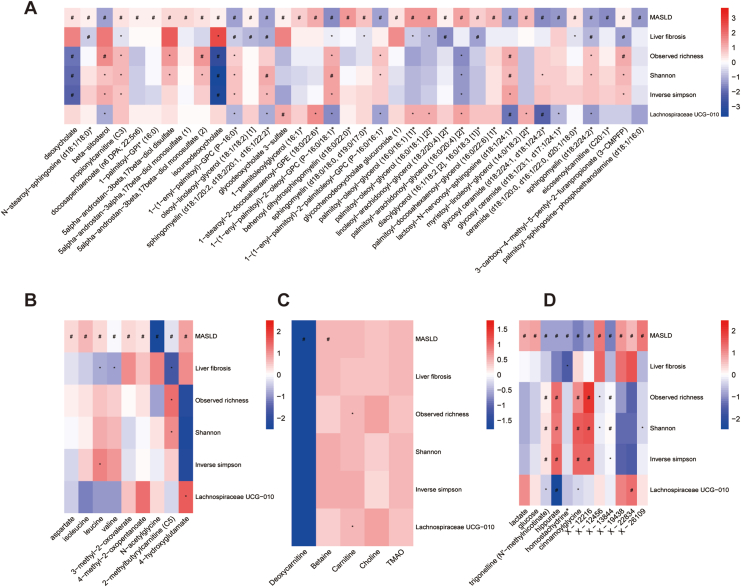
Gut microbiome-derived metabolite profiles of MASLD, liver fibrosis, alpha diversity, and the significant genera. The colours in columns represent the standardized betas of the microbial metabolites as exposure with MASLD and liver fibrosis as various outcomes, in separate multivariable linear and logistic regression models. The association results are after adjustment for age, sex, BMI, smoking status, daily alcohol consumption, and lipid-lower medication use. Moreover, we performed multivariable linear regression, alpha diversity (Shannon index, Inverse Simpson, and Observed Richness), and three significant genera as various outcomes, additionally adjustment for the time in the mail and DNA isolation batch. (A) Lipid profile; (B) Amino acid profile; (C) TMAO and its precursors profile; (D) Other measures. We corrected for multiple testing using Benjamini-Hochberg significant q values (q value<0.05), depicted by #, and nominally significant values (*P* value<.05) by ∗.

Figure 4 A-D shows the gut microbiome-related metabolite profiles of MASLD, liver fibrosis, as well as the associations of these metabolites with alpha diversity, and the significant genera. Notably, 36 microbiome-related lipid metabolites were significantly associated with MASLD (as depicted in Figure 4A). Among these 36 metabolites, the top 2 metabolites (isoursodeoxycholate and deoxycholate) have stronger positively association with MASLD, liver fibrosis and *Lachnospiraceae UCG-010*, also negatively associated with alpha diversity. Figure 4B displays 9 microbiota-associated amino acids metabolites that were significantly associated with MASLD. Liver fibrosis, alpha diversity, and *Lachnospiraceae UCG-010* showed no significant association with plasma levels of TMAO metabolite in this study (Figure 4C), although, Betaine and deoxycarnitine were negatively associated with MASLD (Figure 4C). As shows in Figure 4D, trigonelline (N′-methylnicotinate), hippurate and Cinnamoylglycine metabolic profiles and alpha diversity indices show opposite directions in relation to MASLD. As for hippurate was negatively associated with MASLD and *Lachnospiraceae UCG-010*, whereas it positively associated with alpha diversity indices.

## Discussion

In our research within the population-based Rotterdam Study cohort, we found that participants with MASLD have a lower microbial alpha diversity compared to those without MASLD. The presence of MASLD was associated with a higher relative abundance of *Lachnospiraceae UCG-010* genus. Furthermore, our analysis revealed 160 plasma metabolites associated with MASLD. Of these, 57 metabolites were significantly associated with the gut microbiome at the genus level in individuals with MASLD. Notably, 2 TMAO substrates (betaine and deoxycarnitine) were among the metabolites significantly correlated with the gut microbiome at the genus level in MASLD individuals. Thereby, the associations that we saw for MASLD correlated significantly with gut microbiome and gut microbiota associated metabolites, confirming that the altered gut microbiota and their metabolites affect the presence of MASLD. Additionally, regulation of the composition of gut microbiome may serve as a promising therapeutic strategy for MASLD.

At the genus level, we found 17 genera nominally associated with MASLD after adjusting for quite a board of covariates in full model. Notably, each of these genera exhibited an AUC greater than 0.75, indicating moderate discriminatory power for MASLD. Among them, 5 genera (*Clostridium sensu stricto 1*, *Christensenellaceae R-7 group*, *Dorea*, *Romboutsia*, and *Fusicatenibacter*) were associated with MASLD, which were report on previous research on gut microbiome with hepatic steatosis also embedded in the Rotterdam Study cohort using a different data (16S rRNA sequencing and determined taxonomy using the SILVA version 128 microbial database and high-throughput proton nuclear magnetic resonance metabolomics).^5^ Recently, the abundance of *Clostridium sensu stricto* was identified significantly decreased with the beginning of liver fat accumulation and the development of fibrosis,^23^ which in line with our findings the median abundance of *Clostridium sensu stricto 1* was decreased in participants with MASLD in that study as also found in our study population. Moreover, elevated gut microbiome abundance of *Christensenellaceae* has been associated with reduced visceral adipose tissue and healthier metabolic profile in Italian elderly.^24^ In our study, the median abundance of *Christensenellaceae R-7 group* was decreased in individuals with MASLD.

Recent research has indicated that high alcohol-producing *Klebsiella pneumonia* in the intestinal microbiome could be one of the causes of nonalcoholic fatty liver disease (NAFLD).^25^ Moreover, they suggested that *Klebsiella pneumonia* contributed only little to the overall microbial load signature and was 20 times less abundant than the *Lactobacillaceae*.^25^ In our study, we found that *Klebsiella pneumonia* was present in less than 20% of the samples, so we did not include this genus in the analysis. However, we find the average of the abundance of *Klebsiella pneumonia* was higher in individuals with MASLD than without MASLD, although specific data are not shown.

Additionally, we observed that *Lachnospiraceae UCG-010* was significantly associated with MASLD. An experimental study reported that *Lachnospiraceae UCG-010* was negatively correlated with peroxisome proliferator-activated receptor gamma (PPARγ).^26^ Interestingly, PPARγ is highly expressed in adipose tissue and its activation promotes fat storage in adipocytes and decreases adipose tissue lipolysis thereby decreasing the concentration of fatty acids presented to the liver.^27^ Moreover, the nuclear receptor PPARγ is a ligand activated transcription factor, in turn inhibiting the sentinel transcription factor, nuclear factor-kappa B and thus increasing the threshold for inflammatory responses in general.^28^ Given the key role of PPAR in the transcriptional regulation of glucose and lipid metabolism, PPAR ligands have been investigated also as possible therapeutic agents for fibrotic MASLD.^29^ Therefore, a higher level of *Lachnospiraceae UCG-010* in MASLD individuals, may result in a reduced level of PPARγ and lead to an increase of fatty acids in the liver. It might suggested that the microbiome may predict treatment response on PPARγ. Furthermore, *Lachnospiraceae UCG-010* was reported to be significantly correlated with oxidative stress and lipid metabolism.^26^ The abundance of *Lachnospiraceae UCG-010* effects PPARγ, which warrants further functional and mechanistic studies to investigate its potential links with MASLD.

Our results showed the lipids metabolic profiles of alpha diversity mirrored those of MASLD, having the opposite direction (often significant) with metabolites, which is in agreement with previous studies on gut microbiome, metabolites and metabolic diseases.^5^ A recent study involving 550 Chinese adults with biopsy-proven NAFLD and varying levels of fibrosis found that secondary bile acids levels were significantly increased in NAFLD, especially in those with mild fibrosis.^30^ This aligns with our findings that higher relative abundances of secondary bile acids (eg isoursodeoxycholate, deoxycholate) were observed in MASLD patients.

The 3 BCAAs, valine, leucine, and isoleucine are known to mediate activation of several important hepatic metabolic signaling pathways ranging from insulin signaling to glucose regulation. We observed that BBCAs were positively associated with MASLD. These results are in line with several observational studies, eg a large Finnish population-based study^31^ and a previous study embedded in the Rotterdam Study.^5^ Recent experimental study in animals suggested that, a high BCAAs diet may alleviates MASLD and potentially reprogram the lipidome, improving lipid metabolism.^32^

We also observed significant associations between MASLD and lower levels of betaine and deoxycarnitine (as precursors to TMAO), these results consistent with prior research.^33^ In our study, we did not find a significant association between TMAO and MASLD. These findings may suggest that early steps in the TMAO biosynthesis pathway are more relevant to MASLD pathophysiology than TMAO itself. Alternatively, the lack of association with TMAO in our study could be due to insufficient statistical power, interindividual variability in metabolite measurements, and potential confounding by unmeasured dietary factors. Future studies with larger sample sizes, repeated metabolite measurements, or longitudinal designs may help clarify the potential role of TMAO in the development and progression of MASLD.

Furthermore, we also examined other metabolites, such as lactate, glucose, trigonelline (N′-methylnicotinate), hippurate, homostachydrine∗, and cinnamoylglycine were significantly associated with MASLD. Notedly, hippurate is a normal constituent of the endogenous urinary metabolite profile and has long been associated with hepatic function. Our results confirmed that higher hippurate relative abundances are linked to a lower risk of MASLD, consistent with previous findings that associate increased hippurate with reduced likelihood of metabolic syndrome.^34^ Additionally, our results indicated that increased hippurate relative abundances were associated with higher microbial alpha diversity.

### Limitation of the Study

Our study has some limitations that warrant consideration. First, as this is a cross-sectional observational study, it lacks a longitudinal design. Therefore, we are unable to determine the causal direction of the associations observed. Second, while our integrative approach adds value, we recognize the possibility of sampling bias, and we emphasize the need for larger, independent studies to confirm our findings. Future research incorporating fecal metabolomics may further enhance mechanistic insights by capturing locally produced microbial metabolites. Third, the use of stool samples as a proxy for the gut microbiome might not fully represent the mucosal microbiome, which is more challenging to access. Additionally, MASLD was diagnosed abdominal ultrasound, which, although widely accepted in epidemiological settings, has limited accuracy compared to histological confirmation. Fourth, microbiome profiling was performed using 16S rRNA gene sequencing targeting the V3–V4 regions, which offers limited taxonomic resolution and generally does not allow reliable species-level identification. Consequently, our analyses are restricted to the genus level, which may overlook important species- or strain-specific differences. Fifth, in this study highlight *Lachnospiraceae UCG-010* genus effects *PPARγ* was significant associated with MASLD, although direct functional validation is lacking. Moreover, our reliance on plasma metabolomics may not provide a complete functional assessment of microbial metabolism compared to fecal metabolomics. The Metabolon platform-based metabolomics, while capable of analyzing thousands of samples, still has its limitations when drawing comparisons between samples processed on different runs of the same instrument or other runs from different instruments.^35^

## Conclusion

Taken together, this population-based study indicates a higher abundance of *Lachnospiraceae UCG-010* genus was significant associated with MASLD, several plasma metabolites were significantly correlated with the gut microbiome at the genus level in individuals with MASLD. These findings underscores the potential of integrating gut microbiota analysis and plasma metabolomics with robust liver imaging data within the Rotterdam Study to provide a better understanding of the gut–liver axis in the context of MASLD and liver fibrosis. Although research on microbiome-targeting therapies for MASLD is still in its early stages, much ground-breaking research has been conducted to understand the underlying mechanism of MASLD, many more elements are yet to be investigated. In the future, research is needed to refine microbial-based therapies for personalized treatment of MASLD and should aim at exploring interventions that modulate the composition of the intestinal microbiota.

## Figures and Tables

**Figure S1:**
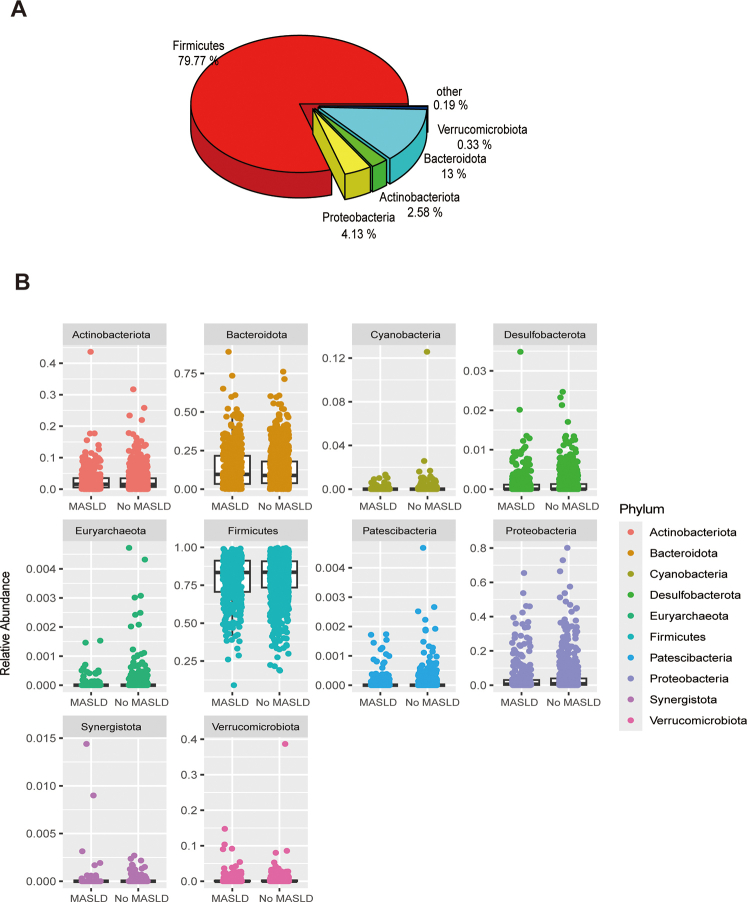


## Supplementary Data

Supplementary data_Final

## References

[1] Younossi Z.A.-O., Golabi P.A.-O., Paik J.A.-O. (2023). The global epidemiology of nonalcoholic fatty liver disease (NAFLD) and nonalcoholic steatohepatitis (NASH): a systematic review. Hepatology.

[2] Feng G., Valenti L., Wong V.W. (2024). Recompensation in cirrhosis: unravelling the evolving natural history of nonalcoholic fatty liver disease. Nat Rev Gastroenterol Hepatol.

[3] Rinella M.E., Lazarus J.V., Ratziu V. (2023). A multisociety Delphi consensus statement on new fatty liver disease nomenclature. J Hepatol.

[4] Ha S., Wong V.W., Zhang X. (2024). Interplay between gut microbiome, host genetic and epigenetic modifications in MASLD and MASLD-related hepatocellular carcinoma. Gut.

[5] Alferink L.A.-O., Radjabzadeh D., Erler N.A.-O. (2021). Microbiomics, metabolomics, predicted metagenomics, and hepatic steatosis in a population-based study of 1,355 adults. Hepatology.

[6] Zhang Y., Wang X., Lin J. (2024). A microbial metabolite inhibits the HIF-2α-ceramide pathway to mediate the beneficial effects of time-restricted feeding on MASH. Cell Metab.

[7] Zeng F., Su X., Liang X. (2024). Gut microbiome features and metabolites in non-alcoholic fatty liver disease among community-dwelling middle-aged and older adults. BMC Med.

[8] Abozaid Y.J., Ayada I., van Kleef L.A. (2024). Circulating metabolites associated with steatotic liver disease and liver enzymes: a multi-platform population-based study. Gastro Hep Adv.

[9] Ikram M.A., Kieboom B.C.T., Brouwer W.P. (2024). The Rotterdam Study. Design update and major findings between 2020 and 2024. Eur J Epidemiol.

[10] Li R., Kurilshikov A., Yang S. (2025). Association between gut microbiome profiles and host metabolic health across the life course: a population-based study. Lancet Reg Health Eur.

[11] Callahan B.J., McMurdie P.J., Rosen M.J. (2016). DADA2: high-resolution sample inference from Illumina amplicon data. Nat Methods.

[12] Quast C., Pruesse E., Yilmaz P. (2013). The SILVA ribosomal RNA gene database project: improved data processing and web-based tools. Nucleic Acids Res.

[13] McMurdie P.J., Holmes S. (2013). phyloseq: an R package for reproducible interactive analysis and graphics of microbiome census data. PLoS One.

[14] Ahmad S., Wu T., Arnold M. (2024). The blood metabolome of cognitive function and brain health in middle-aged adults — influences of genes, gut microbiome, and exposome. medRxiv.

[15] Do K.T., Wahl S., Raffler J. (2018). Characterization of missing values in untargeted MS-based metabolomics data and evaluation of missing data handling strategies. Metabolomics.

[16] Hamaguchi M., Kojima T., Itoh Y. (2007). The severity of ultrasonographic findings in nonalcoholic fatty liver disease reflects the metabolic syndrome and visceral fat accumulation. Official Journal Am Coll Gastroenterol ACG.

[17] Boursier J., Zarski J.P., de Ledinghen V. (2013). Determination of reliability criteria for liver stiffness evaluation by transient elastography. Hepatology.

[18] van Kleef L.A., Francque S.M., Prieto-Ortiz J.E. (2024). Metabolic dysfunction-associated fibrosis 5 (MAF-5) score predicts liver fibrosis risk and outcome in the general population with metabolic dysfunction. Gastroenterology.

[19] Caspersen C.J., Bloemberg B.P., Saris W.H. (1991). The prevalence of selected physical activities and their relation with coronary heart disease risk factors in elderly men: the Zutphen Study, 1985. Am J Epidemiol.

[20] Feunekes G.I., Van Staveren W.A., De Vries J.H. (1993). Relative and biomarker-based validity of a food-frequency questionnaire estimating intake of fats and cholesterol. Am J Clin Nutr.

[21] Goldbohm R.A., van den Brandt P.A., Brants H.A. (1994). Validation of a dietary questionnaire used in a large-scale prospective cohort study on diet and cancer. Eur J Clin Nutr.

[22] Zierer J., Jackson M.A., Kastenmuller G. (2018). The fecal metabolome as a functional readout of the gut microbiome. Nat Genet.

[23] Lanthier N., Rodriguez J., Nachit M. (2021). Microbiota analysis and transient elastography reveal new extra-hepatic components of liver steatosis and fibrosis in obese patients. Sci Rep.

[24] Tavella T., Rampelli S., Guidarelli G. (2021). Elevated gut microbiome abundance of Christensenellaceae, Porphyromonadaceae and Rikenellaceae is associated with reduced visceral adipose tissue and healthier metabolic profile in Italian elderly. Gut Microbes.

[25] Meijnikman A.S., Davids M., Herrema H. (2022). Microbiome-derived ethanol in nonalcoholic fatty liver disease. Nat Med.

[26] Qin S., He Z., Wu Y. (2022). Instant dark tea alleviates hyperlipidaemia in high-fat diet-fed rat: from molecular evidence to redox balance and beyond. Front Nutr.

[27] Esposito K., Ciotola M., Carleo D. (2006). Effect of rosiglitazone on endothelial function and inflammatory markers in patients with the metabolic syndrome. Diabetes Care.

[28] Al-Lahham S.H., Peppelenbosch M.P., Roelofsen H. (2010). Biological effects of propionic acid in humans; metabolism, potential applications and underlying mechanisms. Biochim Biophys Acta.

[29] Liss K.H., Finck B.N. (2017). PPARs and nonalcoholic fatty liver disease. Biochimie.

[30] Liu A.N., Xu C.F., Liu Y.R. (2023). Secondary bile acids improve risk prediction for non-invasive identification of mild liver fibrosis in nonalcoholic fatty liver disease. Aliment Pharmacol Ther.

[31] Kaikkonen J.E., Würtz P., Suomela E. (2017). Metabolic profiling of fatty liver in young and middle-aged adults: cross-sectional and prospective analyses of the Young Finns Study. Hepatology.

[32] Jian H., Li R., Huang X. (2024). Branched-chain amino acids alleviate NAFLD via inhibiting de novo lipogenesis and activating fatty acid β-oxidation in laying hens. Redox Biol.

[33] Zeybel M., Altay O., Arif M. (2021). Combined metabolic activators therapy ameliorates liver fat in nonalcoholic fatty liver disease patients. Mol Syst Biol.

[34] Pallister T., Jackson M.A., Martin T.C. (2017). Hippurate as a metabolomic marker of gut microbiome diversity: modulation by diet and relationship to metabolic syndrome. Sci Rep.

[35] Pietzner M., Stewart I.D., Raffler J. (2021). Plasma metabolites to profile pathways in noncommunicable disease multimorbidity. Nat Med.

